# Development and psychometric properties evaluation of individualized patient education tool: An exploratory sequential mixed-methods study protocol

**DOI:** 10.1371/journal.pone.0361372

**Published:** 2026-10-30

**Authors:** Sevda Gardashkhani, Mansour Ghafourifard, Akram Ghahramanian, Abbas Ebadi, Neda Gilani

**Affiliations:** 1 Department of Medical-Surgical Nursing, School of Nursing and Midwifery, Tabriz University of Medical Sciences, Tabriz, Iran; 2 Student Research Committee, Tabriz University of Medical Sciences, Tabriz, Iran; 3 Medical Education Research Center, Health Management and Safety Promotion Research Institute, Tabriz University of Medical Sciences, Tabriz, Iran; 4 Clinical Research Development Unit of Tabriz Valiasr Hospital, Tabriz University of Medical Sciences, Tabriz, Iran; 5 Nursing Care Research Center, Clinical Sciences Institute, Baqiyatallah University of Medical Sciences, Tehran, Iran; 6 Department of Statistics and Epidemiology, Faculty of Health, Tabriz University of Medical Sciences, Tabriz, Iran; STIKES Wira Medika PPNI Bali: Sekolah Tinggi Ilmu Kesehatan Wira Medika PPNI Bali, INDONESIA

## Abstract

**Background:**

Patient education is an important component of nursing care, influencing treatment outcomes, quality of life, and healthcare costs. However, educational practices are often generalized and may not address patients’ individual needs, health literacy, or socio-cultural contexts. Individualized patient education tailors content to patients’ characteristics, goals, and capabilities, and enhances adherence, satisfaction, and clinical outcomes. Existing tools mainly focus on general aspects of patient education, such as educational quality or provider competencies, and no comprehensive patient-centered tool has been established to assess the individualized nature of patient education. This may constrain systematic assessment and improvement of individualized educational practices. Therefore, this protocol aims to conceptualize individualized patient education, develop a measurement tool, and evaluate its psychometric properties.

**Methods:**

This two-phase exploratory sequential mixed-methods study will be conducted in educational hospitals affiliated with Tabriz University of Medical Sciences, northwest Iran. Phase 1 will involve hybrid concept analysis, including a literature review and directed qualitative study using semi-structured interviews with patients with chronic diseases and their family members until data saturation, followed by directed content analysis to clarify the concept, identify provisional dimensions, and generate initial items, which will be evaluated quantitatively. Phase 2 will assess the tool’s psychometric properties. Face and content validity will be evaluated using qualitative feedback and the Impact Score, Content Validity Ratio, Content Validity Index, and Modified Kappa coefficient. Construct validity will be examined using exploratory and confirmatory factor analyses in SPSS v.24 and AMOS v.24, with independent samples of at least 200 participants per analysis. Reliability will be assessed using Cronbach’s alpha, McDonald’s omega, and test-retest stability. Interpretability and feasibility will also be evaluated.

**Discussion:**

If the tool demonstrates adequate validity and reliability, it may provide a standardized measure for assessing individualized educational interventions and support quality improvement, patient engagement, and evidence-informed health education strategies.

## Introduction

Patient education is a key pillar of healthcare and public health promotion, aimed at enhancing patients’ independence, improving quality of life, and ensuring adherence to treatment [1–3]. Consistent with person-centered care, patient education should respond to patients’ individual needs, characteristics, and health literacy levels rather than be delivered uniformly. This approach, commonly referred to as “individualized education,” aligns with the broader principles of individualized and person-centered care [4,5]. However, healthcare providers, including nurses, may lack awareness of how to individualize patient education and may therefore provide education based primarily on patients’ diagnoses rather than their individual needs [6,7].

In recent decades, individualized patient education has gained increasing attention because of individual differences among patients and the importance of shared decision-making between patients and healthcare teams [8]. Although patient education can be delivered through various formats, traditional approaches are often applied uniformly and may be less effective when they do not account for individual differences, motivation, and health literacy [9–11]. Therefore, considering patients’ unique characteristics and adapting education to their individual needs are essential for effective patient education [12,13]. Evidence indicates that individualized education can improve patients’ participation in treatment decision-making, adherence to treatment, coping, psychological well-being, quality of life, and communication with healthcare providers. It may also reduce hospitalizations and disease recurrence [8,14–17].

Despite these potential benefits, assessing how individualized patient education is in clinical practice remains challenging. Several instruments have been developed to evaluate different aspects of patient education; however, they address constructs that differ from individualized patient education. For example, the Patient Education Implementation Scale focuses primarily on nurses’ implementation of patient education activities [7], whereas the Patient Education Materials Assessment Tool evaluates the quality of educational materials [18]. Similarly, the Patient Education Competence Scale for Registered Nurses in Taiwan assesses nurses’ competence in providing patient education [19], and an instrument developed to assess physician competency in patient education focuses on physicians’ educational competencies [20]. Although these instruments provide valuable information about implementation, educational materials, or professional competencies, they do not specifically assess whether patient education is adapted to individual patients’ needs, characteristics, preferences, level of understanding, and circumstances, particularly from the patient’s perspective.

In the literature, the terms individualized, tailored, and person-centered education are often used interchangeably, although they may emphasize different aspects of patient education. Tailored education typically involves adapting educational content, format, or delivery to individual patient characteristics, often using patient profiles and predefined algorithms or decision rules [21,22]. Person-centered education is a broader approach rooted in person-centered care, focusing on partnership, respect for values and preferences, shared decision-making, and empowerment [23,24]. Individualized patient education, the core concept of this study, has been described in the literature as adapting educational content, timing, methods, and interaction to patients’ individual needs, characteristics, and circumstances [25,26]. However, its specific attributes and dimensions, particularly from the patient’s perspective, remain insufficiently clarified.

This conceptual gap has implications for measurement, as meaningful assessment of individualized patient education requires a clear understanding of its dimensions from the patient’s perspective. Accordingly, the present protocol incorporates hybrid concept analysis as the first phase of instrument development, followed by item generation and psychometric evaluation. The study therefore addresses the following qualitative and quantitative research questions: (1) Qualitative research question: What are the dimensions and characteristics of individualized patient education, and what items can be generated based on the perspectives of patients with chronic diseases and their family members? and (2) Quantitative research question: What are the validity, reliability, interpretability, and feasibility of the developed tool?

### Aim

The main aim of this study is to develop and psychometrically evaluate a tool to measure individualized patient education in the healthcare context. Specifically, the study will: (a) analyze the concept of individualized patient education and determine the dimensions and characteristics of the concept based on the literature review and the perspective of patients with chronic diseases as well as their family members; (b) generate and refine item pools that accurately reflect the dimensions of individualized patient education according to the data extracted from the previous step; and (c) determine the psychometric properties of the tool, including face validity, content validity, construct validity (via exploratory and confirmatory factor analyses), internal consistency, test–retest reliability, interpretability, and feasibility.

## Materials and methods

### Study design

This research is a mixed-methods study with a sequential exploratory design that will be conducted in healthcare centers affiliated with Tabriz University of Medical Sciences in northwest Iran. As shown in Fig 1, the study will be conducted in two phases: Phase 1 (qualitative) and Phase 2 (quantitative). In the qualitative phase, a hybrid concept analysis will be performed to elucidate the concept of individualized patient education. This analysis will identify the concept’s dimensions and characteristics. Then, the items for the measurement tool will be generated, and the initial version of the tool will be designed. In the second phase of the study (quantitative), the psychometric properties of the tool will be evaluated based on relevant Consensus-Based Standards for the Selection of Health Measurement Instruments (COSMIN) recommendations for the measurement properties assessed in this study [27,28]. Specifically, the psychometric characteristics will be examined using face validity, content validity, Item analysis, construct validity (including exploratory factor analysis and confirmatory factor analysis), and reliability will be evaluated through internal consistency and stability. Additionally, the interpretability and feasibility of the tool will be assessed to ensure clinical applicability. Moreover, a connecting strategy will integrate data from the qualitative and quantitative phases [29]. Specifically, findings from the qualitative phase will guide item generation and development of the initial version of the tool, which will then be evaluated using quantitative psychometric methods. The quantitative findings will then be used to confirm, refine, or modify the dimensions and items identified during the qualitative phase. Also, the study reporting will adhere to the Good Reporting of a Mixed Methods Study (GRAMMS) checklist [30].

**Fig 1:**
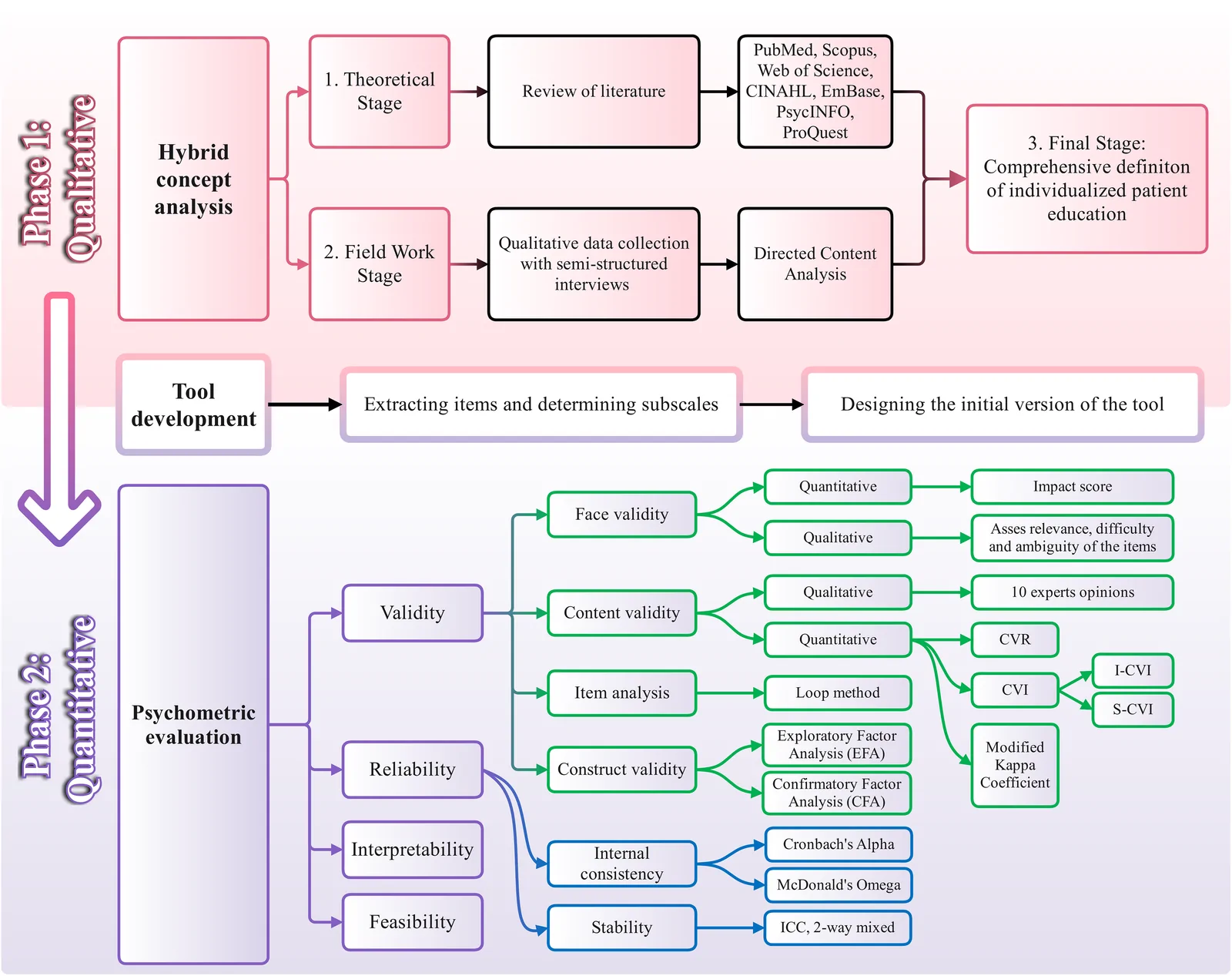
The flowchart of the study.

Philosophically, this study is grounded in the pragmatist paradigm, meaning that the researchers will employ a combination of quantitative and qualitative methods to address the research questions. This ensures suitable depth through qualitative data and certifies precision and generalizability through quantitative data [29].

### Study phases

#### Phase 1: Qualitative phase (hybrid concept analysis).

This phase aims to elucidate the concept of individualized patient education through a literature review and the perspectives of patients with chronic diseases and their family members, leading to the identification of the concept’s dimensions and characteristics and the generation of initial items to develop the measurement tool.

To analyze the concept of individualized patient education, the hybrid concept analysis method developed by Schwartz-Barcott will be employed [31]. The rationale for using this method in this study is that clarifying participants’ perspectives along with a literature review, leads to a better understanding of the concept [32]. As shown in the top section of Fig 1, this method is carried out in three stages, described as follows:

***Stage 1: Theoretical stage.*** The theoretical stage provides the necessary groundwork for a deep analysis of the concept and presents a definition that prepares the researcher for the next stage [31]. To identify the concept’s characteristics and dimensions, scientific databases such as PubMed, Scopus, Web of Science, CINAHL, Embase, PsycINFO, and ProQuest will be searched without time restrictions up to 2026. Papers in English and Persian related to individualized patient education will be selected for full-text review after two researchers independently evaluate titles and abstracts. Inclusion criteria are relevance to individualized patient education and access to the full text. All full-text papers that meet the criteria will be selected for analysis based on the PRISMA 2020 guidelines [33]. Ultimately, the features and factors related to individualized patient education will be extracted and organized into categories and subcategories; this will lead to the formulation of an operational definition of the concept, which will serve as the initial categories in the second stage, i.e., the fieldwork stage.

***Stage 2: Fieldwork stage.*** The goal of the fieldwork stage is to validate and refine the concept developed in the theoretical stage, with particular emphasis on the experiential aspect of individualized patient education. In this stage, the study will use directed qualitative content analysis. This method is particularly suitable because the literature review has already developed an initial theoretical framework. Directed content analysis allows researchers to test and refine existing theoretical categories while remaining open to new insights emerging from participants’ experiences [31,34]. Qualitative data will be collected through face-to-face semi-structured interviews to gain a deeper understanding of the concept from the participants’ perspectives.

***Participants and sampling methods*.** The qualitative phase of the research will be conducted in healthcare centers affiliated with Tabriz University of Medical Sciences. Purposive sampling will be used to select patients and their family members. Initially, key informant participants with rich experience will be selected, such as patients with high educational needs and a history of receiving patient education. Subsequently, based on the progress of the analysis, additional participants will be selected to ensure maximum variation in relevant characteristics. Moreover, to maximize variation, participants will be purposively selected based on personal characteristics (e.g., age, gender), education level (ranging from no formal education to higher education), duration of illness, socioeconomic background, and type of chronic disease (e.g., cardiovascular diseases, diabetes mellitus, chronic respiratory diseases, and other common chronic conditions). This approach will improve the instrument’s generalizability across diverse patient profiles in hospital settings. The study will recruit an initial sample of approximately 20–30 participants, including both patients and family members. The final sample size will be determined based on data saturation. Saturation will be assessed concurrently with data collection and analysis and will be considered achieved when no new codes or categories emerge from subsequent interviews [35]. To confirm saturation, two additional interviews will be conducted after the saturation criterion is met.

***Inclusion and exclusion criteria.*** Table 1 shows the inclusion criteria for participants. Cognitive status will be assessed using the Mini-Mental State Examination (MMSE), which will be administered and scored by a trained researcher (SG) using standardized procedures. A predefined MMSE cut-off score of ≤23 will indicate cognitive impairment, and participants scoring below this threshold will be excluded from the study [36]. Exclusion criteria also include unwillingness to continue participating in the study and a change in the patient’s status to a condition that restricts participation (such as prolonged ICU hospitalization).

**Table 1:** Inclusion criteria for participants.

Inclusion criteria for patients	Inclusion criteria for family members
Having a diagnosis of a chronic disease that requires education	Having an active role in the care and support of the patient
Being at least 18 years old	Being at least 18 years old
Having experience of receiving patient education from healthcare providers	Participation in the patient education process and having a history of receiving information from healthcare providers
No cognitive disorders or severe mental health issues that prevent accurate responses to questions (based on medical records or the researcher’s initial assessment using the MMSE)	No cognitive disorders or severe mental health issues that prevent accurate responses to questions (based on personal statements and the researcher’s initial assessment using the MMSE)

**Note:** MMSE: Mini-Mental Status Examination.

***Data analysis*.** In this stage of the study, directed content analysis, guided by the qualitative content analysis procedures described by Elo and Kyngäs (2008), will be employed to analyze the interview data. This approach was selected because the theoretical stage of the hybrid concept analysis will provide an initial conceptual framework and preliminary categories for examining individualized patient education. The analysis will follow the three phases of qualitative content analysis described by Elo and Kyngäs (2008): preparation, organization, and reporting. During the preparation phase, the unit of analysis will be determined, and the interview transcripts will be read repeatedly to achieve immersion in the data [37].

During the organization phase, a preliminary classification matrix will be developed based on the categories identified during the theoretical stage. The matrix will serve as a guiding rather than rigid coding framework, allowing the interview data to be examined in relation to the predefined categories. The transcripts will then be coded line by line, and meaningful units that correspond to the predefined categories will be assigned to the relevant categories. When relevant data cannot be adequately accommodated within the predefined categories, these data will be retained for further examination. They will inform the refinement or extension of the conceptual categories during the subsequent stage of the hybrid concept analysis [37].

In the final reporting phase, the resulting categories and subcategories will be systematically described and subsequently integrated with the findings of the theoretical stage. This process will contribute to the final conceptualization of individualized patient education and provide the basis for generating items for the measurement tool. MAXQDA 20 software will be used for data management.

***Trustworthiness of qualitative data.*** To enhance the trustworthiness of the data, the strategies proposed by Elo and Kyngäs (2011) will be utilized to ensure rigor in each of the three steps of preparation, organization, and reporting [38]. In the preparation step, the data collection method, sampling strategy, and selection of the appropriate unit of analysis will be adhered to and reported in detail.

In the organization step, more than one researcher will conduct data analysis, and re-coding will be performed to ensure the quality of the classification matrix. Team members will monitor the analysis process, and if disagreements arise, they will hold group discussions. Additionally, the member check method will be used to confirm the data interpretation.

In the reporting step, the characteristics of the participants and the research context will be clearly described to assess the results’ transferability to other contexts. The interviews will be voice-recorded and meticulously analyzed. The concepts and categories will be further clarified through direct quotations, tables, and charts. All steps of the analysis will be reported in detail so that other researchers can achieve similar results.

***Stage 3: Final stage.*** This stage aims to compare and examine the interview results from the fieldwork stage and the literature review from the theoretical stage to achieve the final outcome of the analysis [39]. The codes extracted from the theoretical and fieldwork stages will be compared, and a comprehensive definition of individualized patient education will then be presented. The dimensions and characteristics identified at this stage will be considered provisional and will be confirmed or modified during the quantitative phase through item analysis, exploratory factor analysis, and confirmatory factor analysis.

#### Phase 2: Quantitative phase (Tool development & Psychometric evaluation).

At the beginning of this phase, the initial version of the tool will be developed, as shown in the middle section of Fig 1. Then, the psychometric properties of the developed tool will be evaluated. As shown in the bottom section of Fig 1, this assessment includes face validity, content validity, item analysis, construct validity (exploratory and confirmatory factor analyses), reliability, feasibility, and interpretability.

***Item generation and development of the initial tool.*** To ensure comprehensive coverage of the concept’s dimensions and subscales, a blueprint will be used. The dimensions and subscales will be primarily derived from the operational definition of the concept, resulting in a minimum of three initial items generated for each subscale. To remain consistent with the recommendations and allow flexibility for subsequent refinement and selection, the initial item pool will be larger, i.e., at least twice the number of items anticipated in the final tool [40]. The research team will then thoroughly review the preliminary blueprint. Redundant or highly overlapping items will be eliminated, item wording will be refined for clarity and precision, and each remaining item will be evaluated to ensure it conveys a single, distinct idea. Wording will employ transparent, unbiased language that is grammatically correct and matches the literacy level and comprehension capacity of the target population [41].

***Response format and scoring procedure.*** Concurrently, the team will finalize decisions about the tool’s overall physical structure, response format, and scoring procedures. The measure will be designed as a self-report tool using a Likert-type response scale. The final choice between a 5-point and a 7-point response format will be determined based on pilot study data (n ≥ 30), considering participants’ comprehension of the response options, response distribution and variability, missing responses, and floor and ceiling effects. The response format that demonstrates adequate discrimination between response categories, sufficient response variability, minimal floor and ceiling effects, and greater ease of understanding will be selected.

Each response category will be assigned a numerical score, with higher scores indicating a greater degree of individualized patient education. Item scores will be summed to calculate subscale scores and, where supported by the final factor structure, an overall instrument score. If negatively worded items remain after item refinement and psychometric evaluation, their scores will be reverse-coded before calculating the relevant subscale and total scores.

To facilitate interpretation and comparison across subscales, raw scores will be transformed to a 0–100 scale using the following formula: Transformed Score = [(Raw Score − Lowest Possible Raw Score) / (Highest Possible Raw Score − Lowest Possible Raw Score)] × 100 [27,42]. The same transformation procedure will be applied to each subscale and, where applicable, to the overall instrument score, based on the corresponding minimum and maximum possible raw scores.

***Inclusion and exclusion criteria.*** For the quantitative phase, patients will be eligible to participate if they meet the following criteria: (1) being aged 18 years or older; (2) having a diagnosis of a chronic disease requiring patient education; (3) having previous experience of receiving patient education from healthcare providers; (4) being able to understand and communicate in Persian; and (5) having no cognitive impairment, defined as an MMSE score ≥23 [36]. A trained researcher (SG) will administer and score the MMSE using standardized procedures. Patients with an MMSE score <23 will be excluded.

Participants will be withdrawn from the study if they are unwilling to continue participating or if their clinical condition changes in a way that prevents them from participating, such as prolonged hospitalization in the intensive care unit (ICU).

***Psychometric evaluation***

- **Validity**

***Face validity***

Face validity will be assessed to evaluate the items’ appearance and clarity from participants’ perspective. A combination of quantitative and qualitative methods will be used to assess the tool’s face validity.

In the quantitative face validity, 15 individuals from the target group (patients) will be asked to rate the clarity of each item using a five-point Likert scale (ranging from “completely understandable” to “not understandable at all”). Subsequently, the Impact Score (IS) for each item will be calculated using the following formula:


$$\begin{array}{c} \text{Impact Score }(\text{IS})\;=\text{ Frequency }(\%)\;\times \text{ Comprehensiveness} \end{array}$$

Frequency refers to the number of individuals who rate each item 4 or 5, while comprehensiveness denotes the average score from participants’ responses to that item on the Likert scale. According to the results, items that achieve an IS greater than 1.5 will be deemed suitable. However, items with scores below 1.5 will not be discarded; instead, they will be revised during qualitative face validity based on participants’ feedback.

In the qualitative face validity, semi-structured interviews will be conducted with 15 individuals from the target population (patients). During the qualitative face validity, the relevance to the objective, difficulty of understanding, and ambiguity of the items will be assessed. Then, necessary revisions will be made to ensure that the items are clear, relevant, and comprehensible to the target group [40,43].

***Content validity***

Content validity involves a thorough examination of all aspects of the tool and the ability of the items to reflect the characteristics of the construct being measured. The content validity of the scale will be assessed using both qualitative and quantitative methods [43].

To determine the qualitative content validity of the tool, the opinions of 10 experts, e.g., faculty members from nursing and midwifery schools with academic and research backgrounds in patient education and tool development, as well as experienced nurses and physicians in patient education, will be gathered regarding content coverage, grammatical errors, simplicity, clarity, use of appropriate terminology, and the placement of items within each dimension. Necessary revisions will be made based on the feedback received. The revised items will then be resubmitted to the experts for a second evaluation to confirm their relevance, clarity, and appropriateness.

After confirming the qualitative content validity and implementing the necessary revisions, the quantitative content validity will be evaluated using the Content Validity Ratio (CVR) and the Content Validity Index (CVI).

To determine the Content Validity Ratio (CVR), the opinions of 10 experienced specialists will be utilized. Each of these specialists will rank the items based on the following three-point scale: “essential,” “useful but not essential,” and “not essential.” The CVR for each item will then be calculated using the following formula [44,45]:


$$\begin{array}{c} CVR=\frac{\left(N_{e}-N/\text{2}\right)}{\left(N/\text{2}\right)} \end{array}$$

where $N_{e}$ is the number of specialists who deemed the item essential, and N is the total number of specialists. The results will be compared against the criteria in the Lawshe table [46]. Based on the opinions of 10 specialists, the CVR values greater than or equal to 0.62 will be considered acceptable and the item will be retained [46].

Next, the Content Validity Index (CVI) will be examined. The purpose of the CVI is to determine the relevance of the items to the research objective from the perspective of the specialists. The same 10 specialists who participated in the CVR evaluation will assess each item using a four-point Likert scale (1: not relevant, 2: somewhat relevant, 3: acceptable relevance, 4: completely relevant). The Item Content Validity Index (I-CVI) will be calculated for each item using the following formula [43,47]:


$$\begin{array}{c} I-CVI=\frac{\text{Number of evaluators who scored }\text{3}\text{ or }\text{4}}{\text{Total number of specialists}} \end{array}$$

Decision-making based on I-CVI will proceed as follows: items with a CVI > 0.79 will be retained, those with a CVI between 0.7 and 0.79 will be revised, and items with a CVI < 0.7 will be discarded.

To calculate the overall content validity of the tool (S-CVI), two approaches will be used: Universal Agreement (S-CVI/UA) and Average of Indices (S-CVI/Ave).

Universal Agreement (S-CVI/UA) is calculated by the ratio of the number of items that all evaluators deemed as relevant, to the total number of items. Achieving a value of 80% or higher is considered acceptable, 70–79% requires revision, and less than 70% leads to item removal.

Average of Indices (S-CVI/Ave) is the arithmetic mean of I-CVIs for all of the items. If S-CVI/Ave is greater than 0.9, the overall validity of the tool is deemed adequate [43,47].

To reduce the risk of agreement by chance, the Modified Cohen’s Kappa coefficient ($\kappa^{*}$) is defined as follows [43]:


$$\begin{array}{c} \kappa^{*}=\frac{I-CVI\;-\;p_{c}}{\text{1}-p_{c}}, \end{array}$$

$$\begin{array}{c} p_{c}=\frac{N!}{A!(N-A)!}\times \text{0}.{\text{5}}^{N} \end{array}$$

where $p_{c}$ is the hypothetical probability of chance agreement, N is the number of evaluators, and A is the number of agreements. The $\kappa^{*}$value is interpreted as follows: 0.40$\;\leq \kappa^{*}\leq$ 0.59 corresponds to a fair agreement, 0.60$\;\leq \kappa^{*}\leq$ 0.74 represents good agreement, and $\kappa^{*}>$ 0.74 shows excellent agreement [48,49].

***Item analysis***

The analysis of the items will be conducted before the construct validity. The purpose of this stage is to carry out a pilot study to analyze the items and examine the initial reliability of the tool. A minimum of 30 patients with chronic disease will be selected using a convenience sampling method, and the initial reliability of the tool will be assessed using Cronbach’s alpha. The loop method will be employed, where the correlation of each item with the total score of the tool will be calculated initially. Then items with a correlation of less than 0.2 will be removed, while items with correlations between 0.2 and 0.3 will be reviewed based on their significance for decision-making. Items with a correlation higher than 0.3 will be considered suitable. Additionally, Cronbach’s alpha will be recalculated with the removal of each item to determine the role of each one in the overall coherence of the tool. The correlation between items will also be examined; if the correlation coefficient between two given items exceeds 0.7 and they are semantically similar, one of the items will be removed, or they will be merged [41]. All calculations and analyses in this process will be conducted using IBM SPSS V.24 software.

***Construct validity***

Construct validity helps us understand the extent to which a tool measures a specific construct or concept [43]. In this study, exploratory and confirmatory factor analyses will be utilized to assess the construct validity.

***Exploratory factor analysis.*** The exploratory factor analysis (EFA) will be conducted using an independent sample of 3–10 participants per item or a minimum of 200 participants [40] selected through convenience sampling from patients. Table 2 shows the sample sizes and participant groups for each psychometric assessment stage. The Bartlett’s test of sphericity will be used to assess the suitability of the data for factor analysis. The adequacy of the samples will be evaluated using the Kaiser-Meyer-Olkin (KMO) index. A KMO value close to 1 indicates that the sample is suitable for factor analysis. Factors with eigenvalues greater than unity will be considered as suitable factors, while those with eigenvalues less than one will be disregarded. If the assumption of multivariate normality is met, the maximum likelihood method will be used; otherwise, the principal axis factoring method will be employed to extract the factors. Finally, the items that have communalities greater than 0.2 will be retained, and they will be assigned to the factors only if their factor loadings exceed 0.30 [41].

**Table 2:** Sample sizes and participant groups for each psychometric assessment stage.

Psychometric stage purpose	Sampling method	Participants	Number of participants
Face validity (qualitative and quantitative)	Purposeful	Patients	15
Content validity (qualitative and quantitative)	Purposeful	Health care professionals, Nursing faculty	10
Initial reliability	Convenience	Patients	30
Exploratory factor analysis	Convenience	Patients	At least 200
Confirmatory factor analysis	Convenience	Patients	At least 200
Test-retest reliability	Convenience	Patients	30
Final reliability	Convenience	Patients	At least 200

***Confirmatory factor analysis.*** Subsequently, confirmatory factor analysis (CFA) will be conducted using AMOS V.24 on an independent sample of at least 200 patients, separate from the EFA sample, to validate the factor structure identified through the exploratory factor analysis. The indices listed in Table 3 will be used to evaluate the model fit [50,51]:

**Table 3:** Fit Indices for the Confirmatory Factor Analysis (CFA).

Indices	Acceptable values
χ2/df	< 3
RMSEA	< 0.08
SRMR	< 0.1
CFI	> 0.9
GFI	> 0.9
AGFI	> 0.9
TLI	> 0.9
IFI	> 0.9
PNFI	> 0.5
PCFI	> 0.5

**Note:** χ²/df: Chi-Square to Degrees of Freedom Ratio; RMSEA: Root Mean Square Error of Approximation; SRMR: Standardized Root Mean Square Residual; CFI: Comparative Fit Index; GFI: Goodness-of-Fit Index; AGFI: Adjusted Goodness-of-Fit Index; TLI: Tucker-Lewis Index; IFI: Incremental Fit Index; PNFI: Parsimonious Normed Fit Index; PCFI: Parsimony Comparative Fit Index.

- **Reliability**

Reliability is one of the most important indicators of the quality of a tool, referring to the degree of stability and internal consistency in multiple measurements. In this study, the reliability of the tool for measuring individualized patient education will be assessed using two methods: internal consistency and stability [52].

In the internal consistency method, the correlation of the items with each other, with the subscales, and with the overall tool will be examined to demonstrate that the tool measures a homogeneous construct. For this purpose, Cronbach’s alpha and McDonald’s omega will be utilized, with a cutoff point for acceptable reliability set at 0.7. In the stability method, the tool will be distributed to a minimum of 30 patients who will be selected through simple random sampling, with a two-week interval. The intraclass correlation coefficient (ICC, 2-way mixed) will then be calculated. An ICC coefficient of ≥ 0.75 indicates acceptable stability of the tool [53].

- **Interpretability**

Interpretability describes how meaningful the scores or the changes in the scores of a tool are in a clinical context. As outlined in the COSMIN checklist, interpretability will be evaluated using the following conditions: (1) the proportion of missing or unanswered items to assess completeness of the response; (2) the adequacy of sample size to guarantee the necessary statistical power; (3) distribution of the overall scores to examine the variability in them; (4) floor and ceiling effects, which are significant when more than 15% of the participants obtain the minimum or maximum possible score and (5) minimal important difference (MID), which represents the smallest score change that is perceived as clinically meaningful [27,28]. Since no established external anchor is available for this newly developed tool, MID will be estimated using a distribution-based approach. Specifically, the standard error of measurement (SEM) and 0.5 standard deviation (SD) will be calculated and used to estimate the MID [54].

- **Feasibility**

Feasibility refers to how easily the necessary information can be obtained, accessed, retrieved, and employed for measurement purposes. A tool demonstrates strong feasibility when it accounts for participant characteristics such as age, language, cultural background, cognitive functioning, and overall capabilities of the target population. To promote applicability and broad accessibility, the tool will implement straightforward, plain language prepared at approximately a fourth-grade reading level to accommodate diverse cognitive abilities and educational backgrounds. Feasibility will be evaluated based on the following: (1) response rate, calculated as the percentage of fully completed tools, where a rate of equal or greater than 80% is regarded as acceptable; (2) average completion time, with ≤15 minutes considered feasible; and (3) completion rates stratified by educational attainment (ranging from no formal education to advanced degrees) to assess the generalizability of the tool across diverse groups. Furthermore, aspects like age appropriateness, linguistic clarity, and cultural relevance will be taken into account to enhance overall accessibility [55].

### Study status and timeline

Two pilot semi-structured interviews were conducted in May 2026, following ethical approval, solely to assess the feasibility and clarity of the interview process. These pilot interviews are not considered part of the main study and will not be included in the main study analysis. The main qualitative interviews in Phase 1 and the subsequent quantitative phase in Phase 2 have not yet commenced and will be conducted in accordance with the approved study protocol. No qualitative data analysis, findings integration, or item generation has begun. Data collection for all study phases is anticipated to be completed by April 2027, followed by data analysis in accordance with the study protocol. No main study results have been generated at the time of manuscript submission. The final study findings are expected in mid-2027 and will be disseminated through peer-reviewed publications and presentations at national and international scientific conferences.

### Ethics and dissemination

This research is part of a doctoral project in nursing approved by the Research Ethics Committee of Tabriz University of Medical Sciences. The study received ethical approval, which is coded as “IR.TBZMED.REC.1404.364” on July 21, 2025. It is currently in progress and is expected to last until April 2027. Before commencing the research, a detailed explanation of the study’s purpose and methods will be provided. In both the qualitative and quantitative phases, informed consent will be obtained from all of the participants. For participants involved in the test–retest assessment, the repeated measurement procedure will be explained, and participation will remain voluntary. Participants will be informed that they may withdraw from the repeated assessment at any time without any consequences. All collected data, including sensitive or personal material, will be kept confidential at every stage of the study and stored separately in secure files. The findings will be shared at local, national, and international levels to ensure accessibility to all.

## Discussion

This protocol outlines a sequential exploratory mixed-method study for the development and psychometric evaluation of an individualized patient education tool. This research endeavors to bridge a critical gap in contemporary healthcare by shifting focus from generalized patient education to individualized approaches that cater to the unique needs and characteristics of each patient. The goal is to ensure that the developed tool is valid, reliable, and culturally relevant to the healthcare system.

The current healthcare policies increasingly emphasize tailored interventions, recognizing that a one-size-fits-all approach is often ineffective, particularly in managing chronic diseases like cardiovascular conditions, diabetes, and respiratory disorders [17,56]. While patient education is universally acknowledged as fundamental to improving health outcomes, reducing re-admissions, enhancing self-management, and boosting treatment adherence [2,15,57], current educational methods frequently overlook individual patient differences, leading to inconsistencies in care, suboptimal outcomes, and missed opportunities for meaningful engagement [7,58]. The absence of standardized, patient-perspective tools to assess individualized patient education exacerbates this issue, since most existing tools focus on nurse competencies rather than patient experiences [19,59]. This study directly addresses this deficit by proposing a rigorous methodology to develop a tool that evaluates individualized patient education from the patient’s viewpoint. The innovative use of a hybrid concept analysis in the qualitative phase will ensure that the dimensions of the tool are deeply rooted in both theoretical understanding and the lived experiences of patients and their family members, thereby enhancing the content validity and relevance of the developed items [31,34].

The methodological strength of this study lies in its exploratory sequential mixed-method design, which is particularly suitable for developing tools to assess complex and multifaceted concepts like individualized patient education [29]. The sequential nature, where qualitative findings inform the quantitative psychometric evaluation, ensures that the developed tool is not only statistically sound but also conceptually rich and clinically meaningful. This design allows for an initial in-depth qualitative exploration to define the construct of individualized patient education comprehensively, followed by a robust quantitative psychometric evaluation. The integration of face, content, and construct validity assessments (including exploratory and confirmatory factor analyses), alongside reliability, interpretability, and feasibility evaluations, follows the relevant COSMIN recommendations for the measurement properties investigated in this study [27,28].

Furthermore, the planned evaluation of feasibility and interpretability will be crucial for establishing the utility of the tool in clinical practice, allowing healthcare providers to efficiently administer the tool, accurately interpret the scores, and apply the results in order to tailor the patient education strategies based on their individual needs and preferences. By examining practical aspects such as the time required to complete the tool, ease of understanding for patients, and the proportion of missing responses, feasibility testing will determine whether the tool can be realistically integrated into routine clinical workflows. In addition, assessing interpretability, including score distribution, floor and ceiling effects, and the minimal important change, will ensure that the tool provides meaningful and actionable information for decision-making. These evaluations will ultimately support clinical relevance, ease of use, and sustainability of the tool in real-world settings, which in turn facilitates more individualized, patient-centered education and leads to improvement in the quality of care. The resulting individualized patient education tool will not only serve as a critical tool for assessing and improving the quality of patient education within the Iranian healthcare system. Although the tool may provide a foundation for future adaptation and validation in other cultural and healthcare contexts, additional studies are required before broader applicability can be established.

### Limitations

While this protocol provides a rigorous approach to the development and evaluation of an individualized patient education tool, potential limitations in both its design and execution must be acknowledged. These are considered and mitigated where feasible within the study framework.

First, the qualitative phase uses a hybrid concept analysis to explore perspectives from Iranian patients with chronic diseases and their family members. Although theoretical saturation is targeted, the generalizability of the individualized patient education conceptualizations may be limited to similar cultural and healthcare contexts. Global diversity in patient populations and systems implies that direct tool application elsewhere requires adaptation and validation.

Second, the psychometric phase requires adequate sample sizes for exploratory and confirmatory factor analyses. Recruitment challenges or abrupt dropouts could compromise the statistical power and the generalizability of structural validity findings. Additionally, while comprehensive validity and reliability tests are planned, criterion-related validity against a “gold standard” tool is absent due to the nascent state of the field. This study prioritizes establishing the psychometric properties of the tool; consequently, future research is encouraged to compare the tool with emerging measures.

Third, the use of convenience sampling in the quantitative phase may introduce selection bias and limit the representativeness of participants with chronic diseases. Although efforts will be made to recruit participants with diverse demographic and clinical characteristics, the findings may not fully reflect all patient populations. In addition, as the developed tool relies on patients’ and family members’ perceptions of individualized patient education, response bias may influence participants’ reported experiences. Future studies should further evaluate the tool in different healthcare settings and populations and examine its external validity and responsiveness over time.

## Conclusion

This protocol details a comprehensive sequential exploratory mixed-method study designed to develop and psychometrically evaluate an individualized patient education tool. The study is grounded in the critical understanding that effective patient education must move beyond generic approaches to address the unique needs, preferences, and socio-cultural backgrounds of individual patients. By meticulously defining individualized patient education through a hybrid concept analysis and subsequently employing rigorous psychometric validation methods, this research aims to produce a robust, reliable, and valid tool. The anticipated individualized patient education tool holds significant implications for advancing patient-centered care. In practice, the tool will provide a standardized metric to assess the quality and effectiveness of individualized educational interventions, thereby guiding continuous improvement in nursing and fostering greater patient engagement. Policymakers may benefit from evidence-based insights derived from the application of the tool, particularly within the Iranian healthcare context, while future validation studies in diverse settings will be needed to determine its broader applicability.
